# Immunoregulatory roles of the enteric nervous system in esophageal motility control: mechanisms, advances, and therapeutic potential

**DOI:** 10.3389/fimmu.2026.1987626

**Published:** 2026-10-12

**Authors:** Ao Wang, Yu-Ting Liao, Hong-Jie Cheng, Nai-Wei Zhang, Yan-Mei Cai, Qiao-Yan Zhang, Hai-Xia Zeng, Bo-Yi Jia

**Affiliations:** 1 Department of Spleen and Stomach Diseases, Fangshan Traditional Medical Hospital of Beijing, Beijing, China; 2 Beijing Gulou Hospital of Traditional Chinese Medicine, Beijing, China

**Keywords:** esophageal microbiota, esophageal motility, immune cells, immune regulation, mucosa, muscle, nervous system, neuropeptides

## Abstract

The maintenance of esophageal motility relies on the coordinated actions of the mucosa, nervous system, and muscular layers. The mucosal layer not only serves as a physical barrier but also functions as a sensory interface that detects intraluminal stimuli and contributes to local regulation through the release of bioactive molecules. As the central regulatory component, the nervous system comprises an integrated network formed by the intrinsic enteric nervous system and the extrinsic sympathetic and parasympathetic nervous systems, which collectively integrate afferent signals and coordinate muscular activity. Immune mechanisms also play an important modulatory role in the regulation of esophageal motility. Immune cells, including mast cells, eosinophils, T cells, and macrophages, engage in bidirectional interactions with neuropeptides and the esophageal microbiota, thereby contributing to motility regulation through immune–neural interactions. The muscular layer, composed of striated and smooth muscle, serves as the effector component of motility, with the rhythm and intensity of contraction determining the efficiency of esophageal propulsion and clearance. Accordingly, with esophageal motility as its central focus, this review examines the interactions and coordination among the esophageal mucosa, nervous system, and musculature. Based on a systematic review of recent studies investigating the microscopic regulation of neural and muscular function by neurotransmitters, immune cells, sensory nerve endings, and metabolites derived from the esophageal microbiota, this review elucidates the critical role of the nervous system, particularly the enteric nervous system, in linking mucosal signals to muscular responses, together with its associated immunoregulatory mechanisms. It further explores how these components act synergistically to maintain normal esophageal motility and provides a theoretical basis for pathophysiological research into esophageal motility disorders.

## 1 Introduction

The esophagus is a muscular tube that connects the pharynx to the stomach. Its primary function is to transport a food bolus into the stomach through coordinated, sequential peristaltic contractions while preventing reflux of gastric contents. This function depends on the precise coordination of the mucosa, nervous system, and musculature. When food enters the esophagus, the mucosal layer first detects intraluminal stimuli and initiates signaling; the nervous system then integrates the afferent information and generates motor commands; finally, the muscular layer contracts to produce propulsive force. These three components are closely interconnected and together generate esophageal peristalsis and coordinated sphincter opening.

Within this regulatory pathway, the nervous system serves as a bridge by transmitting signals detected by the mucosa, integrating this information, and conveying motor commands to the muscular layer to generate propulsive force, thereby coordinating esophageal peristalsis with sphincter relaxation. Specifically, the enteric nervous system within the esophageal wall regulates local reflexes and motor patterns, whereas extrinsic autonomic pathways, including the vagus nerve, provide higher-order regulation and sensory input. Precise coordination between neural and muscular activity therefore constitutes a central mechanism underlying esophageal motility. Dysfunction at any point along this pathway may lead to esophageal motor disorders, including gastroesophageal reflux disease (GERD), achalasia (AC), diffuse esophageal spasm, esophagogastric junction outflow obstruction (EGJOO), and hypercontractile esophagus. Importantly, although these disorders may all involve impaired lower esophageal sphincter (LES) relaxation and/or abnormal esophageal propulsion, their underlying mechanisms differ substantially. AC is characterized primarily by the loss of inhibitory nitrergic neurons in the myenteric plexus and represents a structural and irreversible lesion. In contrast, GERD is characterized mainly by a functional imbalance involving excessive nitric oxide (NO)-mediated inhibition and reduced cholinergic excitation, generally without irreversible neuronal loss. Thus, neuronal loss, dysregulated neural control, or abnormal muscular responses to neural signals can ultimately manifest as distinct forms of esophageal motor dysfunction. These disorders have diverse clinical manifestations, most commonly including dysphagia, chest pain, regurgitation, and heartburn (1), and may substantially impair quality of life in severe cases.

With advances in molecular biology, neuroscience, and electrophysiological techniques, our understanding of the regulation of esophageal motility has evolved from the organ level to the level of molecular networks. Traditional studies have primarily focused on the central regulation of swallowing, whereas relatively limited attention has been paid to the local reflex circuits formed by the myenteric and submucosal plexuses within the esophageal wall. These local circuits represent a critical node through which mucosal immune signals influence neuromuscular output. Gastrointestinal macrophages can be classified into mucosal lamina propria macrophages and muscularis macrophages (MMφ) (2). The latter are located around the myenteric plexus, participate in the regulation of smooth muscle motility, and exhibit a bidirectional interdependence with enteric neurons. Immune events occurring in the mucosal layer can indirectly affect myenteric plexus function by altering the activation state of MMφ (3). Thus, neural regulation of the esophagus and mucosal immunity do not function independently but rather constitute a continuous and integrated regulatory system. The mechanisms involving mast cells, eosinophils, T cells, and macrophages discussed in this review can all be considered manifestations of pathological activation of this immune–neural axis at different levels. On this basis, this review systematically examines the mechanisms underlying the development and progression of esophageal motility disorders from the perspective of muscular innervation by the neural plexuses within the esophageal wall and explores the novel insights that these mechanisms may provide for clinical diagnosis and treatment (**Figure 1**).

**Figure 1:**
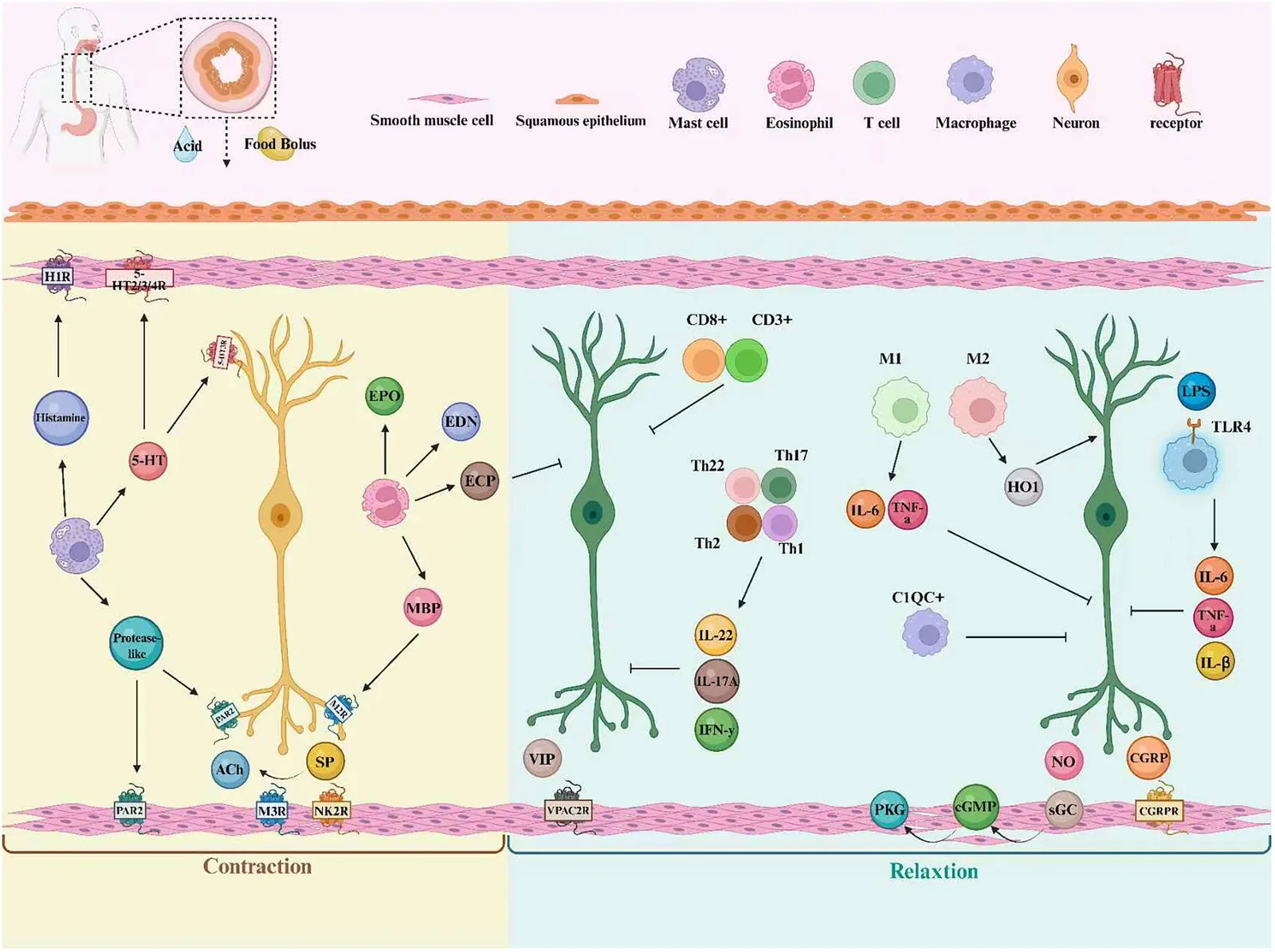
Immunoregulatory roles of the enteric nervous system in esophageal motility control: mechanisms, advances, and therapeutic potential. Figure created with BioRender.com. HIR, Histamine Receptor; 5-HT2/3/4R, 5-Hydroxytryptamine (Serotonin) Receptors 2, 3, and 4; PAR2, Protease-Activated Receptor 2; M2/3R, Muscarinic Acetylcholine Receptor M2/3; NK2R, Neurokinin 2 Receptor; TLR4, Toll-Like Receptor 4; VPAC2R, Vasoactive Intestinal Peptide Receptor 2; CGRPR, Calcitonin Gene-Related Peptide Receptor; CD8^+^/CD3^+^, T cell surface differentiation antigen markers; C1QC^+^, Complement C1q C-chain-positive macrophages; M1/M2, Macrophage polarization phenotypes (M1 pro-inflammatory, M2 anti-inflammatory); 5-HT, 5-Hydroxytryptamine; ACh, Acetylcholine; SP, Substance P; EPO, Eosinophil Peroxidase; EDN, Eosinophil-Derived Neurotoxin; ECP, Eosinophil Cationic Protein; MBP, Major Basic Protein; VIP, Vasoactive Intestinal Peptide; LPS, Lipopolysaccharide; IL-6, Interleukin-6; TNF-α, Tumor Necrosis Factor-alpha; IL-1β, Interleukin-1 beta; IL-22, Interleukin-22; IL-17A, Interleukin-17A; IFN-γ, Interferon-gamma; HO1, Heme Oxygenase-1; NO, Nitric Oxide; CGRP, Calcitonin Gene-Related Peptide; PKG, Protein Kinase G; cGMP, Cyclic Guanosine Monophosphate; sGC, Soluble Guanylate Cyclase; Th1, T helper 1 cell; Th2, T helper 2 cell; Th17, T helper 17 cell; Th22, T helper 22 cell.

## 2 Cellular composition of the esophageal mucosa

The esophageal wall consists of the mucosa, submucosa, muscular layer, and adventitia, which together contribute to food transport and tissue protection (4).

The mucosa is one of the most important functional layers of the esophagus and consists of nonkeratinized stratified squamous epithelium, the lamina propria, and the muscularis mucosae. The epithelial layer provides protection by forming a mechanical barrier against friction generated during food passage (5). The lamina propria contains fibroblasts and abundant immune cells, including lymphocytes, plasma cells, and macrophages, which contribute to mucosal immune defense (6). The muscularis mucosae is composed of smooth muscle and helps regulate the configuration of the esophageal lumen. The submucosa contributes to nutrient supply and the local regulation of motility. The muscular layer consists of an inner circular layer and an outer longitudinal layer, and the orientation of these muscle fibers is closely associated with peristaltic transport of food. Anatomically, the upper esophagus is composed predominantly of striated muscle, the middle portion contains a mixture of striated and smooth muscle, and the lower portion is composed predominantly of smooth muscle (7). The esophagus lacks a serosal covering, which facilitates early invasion of adjacent organs by esophageal cancer and is therefore of considerable clinical importance (8).

## 3 Innervation of the esophagus

### 3.1 Dual afferent pathways of esophageal sensation and their descending modulation

From an afferent sensory perspective, mechanoreceptors, chemoreceptors, and thermoreceptors are distributed throughout the esophageal wall, and signals generated by these receptors are transmitted through two major pathways (9, 10). The first is the vagal pathway. The cell bodies of primary afferent neurons are located in the nodose ganglion, and their central processes terminate in the nucleus tractus solitarius of the medulla. This pathway mainly conveys non-nociceptive physiological sensations, such as distension and the passage of luminal contents, as well as dull discomfort. The second is the spinal pathway. The cell bodies of primary afferent neurons are located in the dorsal root ganglia at the T1–T6 spinal levels, and their fibers reach the spinal dorsal horn through the sympathetic chain. This pathway mainly transmits sharp pain, burning pain, and high-intensity chemical stimuli, such as the heartburn sensation caused by gastric acid reflux (11). Signals from these two pathways undergo extensive integration in the nucleus tractus solitarius and spinal dorsal horn and are also subject to descending modulation from higher regions of the central nervous system, which can enhance or suppress local responses.

### 3.2 Segmental organization of esophageal innervation

The cervical esophagus consists predominantly of striated muscle and is controlled by somatic motor innervation. The cell bodies of its motor neurons are located mainly in the nucleus ambiguus of the brainstem. Their myelinated axons travel within the vagus nerve and project directly and continuously to the esophageal musculature, where they form typical motor endplates (12–14). At the neuromuscular junction, acetylcholine (ACh) released from motor nerve terminals acts on nicotinic cholinergic receptors to induce contraction. Therefore, coordinated propulsion in the striated-muscle segment of the esophagus depends heavily on centrally programmed, sequential discharge of motor neuron populations in the nucleus ambiguus.

The thoracic esophagus can be further divided into upper, middle, and lower segments. The upper thoracic segment remains predominantly composed of striated muscle (15), with relatively sparse intramural neural plexuses.

The middle thoracic segment represents a transitional zone from striated to smooth muscle. Vagal fibers become more abundant and begin to form the anterior and posterior esophageal plexuses. The myenteric plexus also begins to appear between the circular and longitudinal muscle layers, although its density remains relatively low and its structural organization is not yet fully developed.

The lower thoracic segment consists predominantly of smooth muscle. The myenteric plexus is particularly dense and well developed in this region (16) and contains excitatory cholinergic neurons as well as inhibitory nitrergic and peptidergic neurons. The submucosal plexus is also present but is relatively sparse (17) and mainly regulates glandular secretion and local blood flow.

In the abdominal esophagus, extending from the esophageal hiatus to the cardia, the dense distribution of the myenteric plexus continues from the lower thoracic segment (16). Inhibitory neurons are particularly abundant in the LES region, whereas the submucosal plexus gradually becomes continuous with the gastric submucosal plexus. These intramural neural plexuses participate in the local reflex regulation of gastroesophageal reflux.

### 3.3 Layer-specific characteristics of esophageal wall innervation

Innervation of the esophageal wall exhibits a distinct layered distribution. Extrinsic nerves, including parasympathetic fibers derived mainly from the vagus nerve and sympathetic fibers originating from the thoracic sympathetic trunk, interact with intrinsic nerves of the intramural neural plexuses, namely the submucosal and myenteric plexuses. Together, they form a complex regulatory network across the different layers of the esophageal wall and jointly regulate motility, sensation, and glandular secretion. The adventitia is the initial site through which nerve trunks enter the esophageal wall. Branches of the vagal and sympathetic nerves first anastomose within the mediastinal connective tissue outside the adventitia to form a periesophageal neural plexus. After penetrating the adventitia, these fibers progressively branch and extend toward deeper layers. Motor-related fibers primarily enter the muscular layer and synapse with, or otherwise establish connections with, neurons in the myenteric plexus located between the longitudinal and circular muscle layers, thereby regulating contraction and relaxation of the esophageal musculature. Fibers associated with sensation and secretion extend further inward into the submucosa, where they contribute to the formation of the submucosal plexus and ultimately give rise to terminal endings within the mucosa, thereby mediating sensory signal transduction and the regulation of glandular secretion (18).

#### 3.3.1 Myenteric plexus

The muscular layer exhibits the most complex neural innervation within the esophageal wall and plays a critical role in coordinating peristalsis and sphincter function. Early morphological studies in Taiwanese monkeys demonstrated the presence of a myenteric plexus between the inner circular and outer longitudinal muscle layers (19). This plexus is poorly developed in the upper esophagus but becomes substantially more developed in the middle and lower segments. Its ganglion cells consisted mainly of Dogiel type I cells and apolar or immature-type cells, whereas Dogiel type II cells were not identified. The distribution and innervation pattern of the myenteric plexus are closely related to the gradual transition in esophageal muscle fiber type from the upper to the lower esophagus. Extrinsic parasympathetic innervation of striated muscle in the upper esophagus originates mainly from motor neurons in the rostral medullary nucleus ambiguus, whose axons travel through the vagus nerve and terminate directly at motor endplates on muscle fibers. In contrast, parasympathetic innervation of smooth muscle in the lower esophagus originates from the dorsal motor nucleus of the vagus, with preganglionic fibers forming synapses with postganglionic neurons in the myenteric plexus of the esophageal wall (20). Thus, most vagal efferent fibers are preganglionic fibers that primarily target neurons of the myenteric plexus, which subsequently innervate esophageal smooth muscle.

#### 3.3.2 Submucosal plexus

The submucosa contains the submucosal plexus, which is more developed in the upper esophagus than in the lower esophagus. Its neuronal cells are usually morphologically immature, although occasional Dogiel type I cells are present. Unbranched and simply branched sensory nerve endings are also distributed within the submucosa and lamina propria (19). These structures participate in the regulation of glandular secretion and the reception of sensory signals and collectively contribute to the complex physiological functions of the esophagus.

### 3.4 Dual mechanisms of esophageal motor regulation: parallel central mediation and local reflexes

Neural regulation of esophageal motility involves two parallel and interconnected mechanisms (21, 22). The first is a centrally mediated mechanism in which the brainstem swallowing pattern generator integrates afferent signals from the nucleus tractus solitarius and generates efferent output through vagal motor neurons. This mechanism primarily controls complex coordinated movements, such as swallowing and peristalsis of the striated esophagus. It originates in the swallowing center of the medulla. Parasympathetic preganglionic neurons arising from the dorsal motor nucleus of the vagus send axons that converge to form vagal efferent fibers. These fibers descend through the neck and thorax and, after entering the esophageal wall, form synaptic connections with postganglionic neurons in the myenteric plexus. Vagal preganglionic terminals release ACh, which acts on neuronal nicotinic receptors on postganglionic neurons of the myenteric plexus. This activates either excitatory cholinergic or inhibitory nitrergic postganglionic neurons, resulting in smooth muscle contraction or relaxation, respectively.

The second mechanism is a local neural reflex mediated by coordinated activity between peripheral primary afferent neurons and intrinsic neurons of the myenteric plexus (23). In this pathway, activation of mucosal receptors or mechanoreceptors excites peripheral afferent neurons that communicate with intramural effector neurons. This pathway contributes to the fine regulation of esophageal muscle contraction and mediates functions such as mucosal protection and the sensing of mechanical stress. The local reflex arc comprises three neuronal components: primary sensory neurons, interneurons, and motor neurons. When local, non-swallow-related distension or chemical stimulation occurs in the esophagus, mechanoreceptors, including stretch receptors, or chemoreceptors in the submucosa and muscular layer are activated and generate action potentials. Their axon terminals form synaptic connections with interneurons in the myenteric plexus. Following rapid local integration of these signals, the interneurons activate motor neurons within the same or adjacent segments, producing contraction or relaxation. Although the role of central pathways in the regulation of esophageal function has been extensively investigated, alterations in local reflexes under physiological and pathological conditions remain insufficiently characterized.

## 4 Neural regulation and esophageal motility disorders

### 4.1 Myenteric plexus and motility disorders

The esophageal wall contains a complete enteric nervous system, in which the myenteric plexus serves as a major regulatory center for smooth muscle motility. Effective esophageal peristaltic propulsion depends on coordinated activity between excitatory and inhibitory neurons. Inhibitory neurons are activated before excitatory neurons, allowing distal esophageal smooth muscle to relax in advance and thereby creating a pathway for passage of the food bolus.

#### 4.1.1 Excitatory cholinergic pathways and impaired peristalsis

The cholinergic system serves as the direct source of propulsive force. ACh is the principal effector neurotransmitter responsible for contraction of esophageal smooth muscle. During swallowing, activation of vagal preganglionic fibers induces firing of cholinergic neurons in the myenteric plexus. The released ACh then acts on M3 muscarinic acetylcholine receptors on smooth muscle cells, triggering intracellular Ca^2+^ mobilization and myofilament interaction and thereby generating contraction.

Swallow-induced peristalsis in humans depends predominantly on cholinergic pathways. In the smooth-muscle segment of the esophagus, swallow-induced peristalsis is mainly driven by centrally programmed sequential activation of intrinsic excitatory cholinergic neurons. Thus, central and peripheral regulatory mechanisms converge on cholinergic neurons as a common final pathway (24).

Reduced excitatory neuronal function is a major cause of impaired esophageal body peristalsis. An authoritative review (25) reported that experimental studies in both animals and humans have shown that, during esophagitis, an influx of pro-inflammatory cytokines into the esophageal wall suppresses ACh release from excitatory neurons of the myenteric plexus to the circular muscle layer. These findings indicate that impaired esophageal body peristalsis is largely attributable to reduced excitatory neuronal function. Experimental evidence supporting this mechanism was provided by Cao et al. (26) using a feline model of reflux esophagitis (RE). Both the amplitude and propagation velocity of contraction waves in the distal esophagus were significantly reduced, while choline acetyltransferase (ChAT) activity in esophageal muscle tissue was significantly lower than that in normal controls. This reduction was particularly pronounced in the distal esophagus.

#### 4.1.2 Inhibitory nitrergic pathways and abnormal LES relaxation

The nitrergic system provides the molecular basis for the inhibitory front and LES relaxation. Swallow-induced LES relaxation and anticipatory relaxation occurring ahead of the peristaltic contraction in the esophageal body both depend on NO released from inhibitory non-adrenergic, non-cholinergic (NANC) neurons.

Current evidence indicates that NO is the most important postganglionic neurotransmitter responsible for LES relaxation. NO is synthesized by neuronal nitric oxide synthase (nNOS), which is expressed in neurons of the myenteric plexus and colocalizes with vasoactive intestinal peptide (VIP). VIP may serve as a second inhibitory neurotransmitter in the distal esophagus and LES. Both *in vitro* and *in vivo* studies have demonstrated that nNOS inhibitors can block nerve-mediated LES relaxation (27).

Hyperactivity of inhibitory neurons is a major mechanism underlying abnormal LES relaxation. Yu et al. (28) found that nNOS- and VIP-positive nerve fibers in the esophageal muscular layer were more numerous and thicker in patients with RE than in healthy controls and exhibited stronger staining intensity. These findings suggest abnormal alterations in nitrergic and VIPergic inhibitory innervation of the esophageal wall and indicate that enhanced inhibitory neuronal activity may contribute to abnormal regulation of esophageal motility. Tomita et al. (29) provided further evidence supporting this mechanism by comparing myenteric neural function in the LES between patients with gastric cancer with GERD and those without GERD. Although cholinergic innervation of the LES was reduced in patients with GERD, a more prominent finding was the marked enhancement of NANC inhibitory neural activity. NO mediates sphincter relaxation induced by NANC inhibitory nerves, and this relaxation response was further enhanced in patients with GERD. Taken together, an imbalance between cholinergic and NANC inhibitory neural activity, particularly enhanced NO-mediated NANC inhibition in combination with impaired cholinergic function, represents an important neural mechanism contributing to reduced LES pressure in GERD.

### 4.2 Parasympathetic and vagal regulation in esophageal motility disorders

Parasympathetic and vagal innervation also contributes to the regulation of esophageal motility and is involved in the development and progression of several esophageal disorders.

Cunningham et al. (30) assessed autonomic nervous system function using cardiovascular reflex tests together with measurements of esophageal transit and motility, including delayed transit. They found that 44% of patients with GERD exhibited autonomic dysfunction predominantly involving the parasympathetic nervous system. This dysfunction was significantly associated with a higher proportion of simultaneous esophageal contractions and delayed esophageal transit, suggesting that impaired parasympathetic function may contribute to the pathogenesis of GERD by disrupting peristalsis of the esophageal body.

Vagal dysfunction is not limited to GERD. In a clinical comparative study, Ohlsson et al. (31) found evidence of impaired vagal function in patients with AC. The E/I ratio reflecting vagal activity was significantly lower in patients with AC than in healthy controls. These findings suggest that neural abnormalities in AC are not restricted to degenerative changes within the esophageal wall but may also involve more widespread functional impairment of the extrinsic vagal nervous system. In particular, vagal efferent dysfunction may directly contribute to reduced contractile activity of distal esophageal smooth muscle and impaired LES relaxation.

## 5 Immune cells exert particularly prominent effects on efferent neural control of esophageal motility

### 5.1 Mast cell degranulation mediates esophageal neuromotor dysfunction

Recent studies have provided important insights into this process from the perspective of neurogenic regulation of motility. A prospective study using high-resolution esophageal manometry provided functional evidence for mast cell-associated alterations in neural regulation. Park et al. (32) found that mast cell infiltration in the esophageal mucosa of patients with non-cardiac chest pain was significantly associated with both distal esophageal segmental contraction volume and the distal-to-proximal segmental contraction ratio. Mast cells are distributed mainly in the vascular papillae and lamina propria and are also abundant in the muscularis mucosae and muscularis propria, whereas relatively few are present within the epithelium (33). Following IgE cross-linking or stimulation by substance P (SP), mast cells undergo degranulation and release a variety of mediators (34).

#### 5.1.1 Mast cell infiltration is associated with reductions in ICCs, nNOS-positive cells, and glial cells

Several studies (35–37) have reported significantly increased mast cell infiltration in the LES of patients with esophageal motility disorders, including AC and eosinophilic esophagitis (EoE). This increase is closely associated with reductions in interstitial cells of Cajal (ICCs), nNOS-positive neurons, and S-100-positive glial cells, suggesting that mast cells may represent an important link between neural regulation and muscular motility. Nelson et al. (35) noted that pro-inflammatory and cytotoxic substances released during mast cell degranulation have been shown to damage neurons in disorders of the central nervous system. They therefore proposed that mediators released from degranulating mast cells may exert neurotoxic effects, disrupt motor regulation, and damage myenteric neurons, thereby disturbing neuro-esophageal motor interactions and potentially contributing to disease development and progression.

#### 5.1.2 Mast cell-derived mediators influence motility through neural regulation

Mast cell-derived mediators act through multiple neural receptors. Tryptase is released in large quantities from degranulating mast cells (38). Nelson et al. (39) found that mast cell degranulation in the LES muscular layer of patients with AC or EGJOO involved multiple anatomical compartments, including the muscularis propria and both the circular and longitudinal muscle layers. Large numbers of tryptase-positive granules were released into the endomysium and perimysium. In some regions, the granules were diffusely distributed, whereas in others they remained clustered around individual mast cells. Notably, mast cell degranulation was particularly prominent around the myenteric plexus between the circular and longitudinal muscle layers. Granules were distributed not only around nerve sheaths but also frequently penetrated into neural tissue.

Using isolated longitudinal smooth muscle strips from the opossum esophagus, Liu et al. (40) demonstrated that these proteases can cleave and activate protease-activated receptor 2 (PAR2) on sensory nerve axons within the esophageal wall. Through an axon reflex, this activation induces local release of SP from sensory nerve terminals. SP subsequently acts on neurokinin-2 receptors (NK2R) on smooth muscle cells, thereby inducing contraction of esophageal longitudinal smooth muscle. This contractile response depends on capsaicin-sensitive sensory nerves. Bai et al. (41) similarly found in porcine esophageal body tissue that trypsin induced biphasic relaxation and contraction of the circular muscle through PAR2, transient receptor potential vanilloid 1 (TRPV1), and neurokinin receptor pathways. These findings further hint that tryptase released during mast cell degranulation may indirectly regulate esophageal smooth muscle through neural mechanisms.

Nelson et al. (35) further proposed that neurotoxic mediators released during mast cell degranulation may not only damage myenteric neurons but also directly activate contractile pathways in esophageal smooth muscle through multiple signaling mechanisms or indirectly enhance smooth muscle contraction by altering cholinergic neural activity. For example, LES smooth muscle cells express PAR2, which can be activated by tryptase-mediated cleavage, and tryptase is abundantly released from degranulating mast cells (38). Mast cell degranulation also releases histamine, which can enhance basal LES tone. In human LES specimens, histamine induces LES contraction through H1 receptors, suggesting that mast cell degranulation may increase the myogenic or neurogenic tone of the antireflux barrier through histaminergic signaling (42). Tryptase and histamine may therefore act synergistically to enhance contractile responses.

Studies have also shown that 5-hydroxytryptamine, or serotonin (5-HT), can be synthesized and stored in human mast cells (43). Mast cell-derived 5-HT may bind to 5-HT3 receptors on cholinergic neurons of the enteric nervous system, thereby triggering the release of excitatory transmitters such as ACh and SP, which subsequently act on M3 receptors on smooth muscle to induce contraction. In an *ex vivo* study of porcine LES circular muscle strips, Lecea et al. (44) observed that 5-HT induced concentration-dependent LES contraction. This response exhibited neurogenic characteristics involving both cholinergic and non-cholinergic transmitters. Oh et al. (45) found that, in feline esophageal smooth muscle cells, 5-HT binds to 5-HT2, 5-HT3, and 5-HT4 receptors and activates phospholipase C (PLC), leading to activation of Ca^2+^/calmodulin-dependent myosin light-chain kinase (MLCK), phosphorylation of the 20-kDa myosin light chain (MLC20), and subsequent cellular contraction. Thus, both neural and direct smooth muscle pathways may contribute to 5-HT-induced contraction.

Under persistent or excessive inflammatory stimulation, these effects may become more pronounced. Sustained mast cell degranulation releases large amounts of inflammatory and cytotoxic substances capable of damaging neurons. If these mediators affect the esophageal myenteric plexus, they may damage or eliminate inhibitory neurons involved in the control of esophageal motility. When this occurs in conjunction with excessive activation of excitatory neurons, the normal coordination between esophageal relaxation and contraction may be disrupted, and spastic contractions of the esophageal body may develop. Such abnormalities are characteristic manifestations of AC and represent features of severe esophageal motility dysfunction. However, studies in this area remain limited, and the underlying mechanisms require further investigation.

### 5.2 Eosinophil-derived neurotoxic proteins mediate esophageal neural injury and motility dysfunction

Eosinophils are generally absent from the healthy esophagus (33); therefore, their accumulation in esophageal tissue usually indicates a pathological condition. In patients with EoE, eosinophils predominantly infiltrate the esophageal mucosal epithelium, which is a classic diagnostic feature of the disease (46).

Studies of deeper tissue layers have shown that, in deep esophageal biopsy specimens from patients with EoE, eosinophils accumulate around neural cells associated with VIP signaling (47). Studies of motility disorders such as AC have also demonstrated that eosinophils can infiltrate deeper into the muscularis propria and localize adjacent to neurons within the muscular layer (48).

#### 5.2.1 Eosinophil infiltration is associated with neural injury

Eosinophils are pleiotropic leukocytes involved in the initiation and propagation of inflammatory responses. Following activation and degranulation, they release cytotoxic granules containing cationic proteins that can cause neural tissue injury and dysfunction (49). Dense eosinophilic infiltration has been identified in the esophageal muscular layer of patients with hypercontractile disorders (50). In a pathological study, Jin et al. (51) found varying degrees of activated eosinophil infiltration in the middle and distal esophagus and LES of patients with AC, accompanied by a marked reduction or complete loss of ganglion cells in the myenteric plexus. These findings suggest that activated eosinophils may contribute to esophageal motility disorders by inducing injury to intramural esophageal neurons.

#### 5.2.2 Eosinophil-derived products influence motility through neural regulation

Eosinophils can influence esophageal motility through multiple pathways and may produce either spastic or hypercontractile activity or, conversely, hypomotility or absent peristalsis, depending on the predominant mechanism. By releasing neurotoxic proteins that act on intramural neural plexuses, eosinophils can alter the local balance between excitation and inhibition, thereby affecting the amplitude and coordination of contractile waves. This may represent an important mechanism underlying esophageal motility dysfunction. Major eosinophil-derived products include major basic protein (MBP), eosinophil cationic protein (ECP), eosinophil peroxidase (EPO), and eosinophil-derived neurotoxin (EDN) (52).

Fredens et al. (53) reported a case of secondary AC associated with gastric cancer in which extensive activated eosinophilic infiltration was present throughout the esophageal wall, particularly around the myenteric plexus and between muscle fibers. Eosinophils underwent degranulation and released ECP, accompanied by a profound reduction in VIP-, SP-, and acetylcholinesterase (AChE)-positive nerve fibers. These findings suggest that toxic products released during eosinophil degranulation may damage the esophageal myenteric plexus and intramural nerves and may be associated with loss of esophageal innervation and the typical motor abnormalities of AC, including absent esophageal peristalsis and impaired LES relaxation. The authors further proposed that such neurotoxic effects might damage intramural vagal branches, induce Wallerian degeneration, and potentially even affect central neural nuclei. Subsequent studies further supported this concept (54), showing that mediators released from degranulating eosinophils can damage the esophageal myenteric plexus involved in peristalsis and LES relaxation and may therefore contribute to impaired LES relaxation and irreversible esophageal motility dysfunction.

Visaggi et al. (55) noted that eosinophil-derived products can induce axonal necrosis and impair effective neurotransmitter transmission. Tottrup et al. (56) detected activated ECP deposition in distal esophageal tissue from patients with AC. At the same time, esophageal specimens commonly exhibited injury to the myenteric plexus, characterized by loss of VIPergic and SP-containing peptidergic innervation and a marked reduction in ganglion cell numbers. Jia et al. (57) examined sling and clasp fibers of the LES in patients undergoing esophagectomy for esophageal cancer and found that human LES tissue expressed only VPAC2 receptor mRNA and protein, suggesting that VIP induces LES relaxation through VPAC2 receptors. Direct injury by ECP to peptidergic nerves and ganglion cells within the myenteric plexus may therefore result in incomplete swallow-induced LES relaxation and loss of esophageal body peristalsis, ultimately producing smooth muscle motor dysfunction. Thus, eosinophil-derived components may promote AC through neurotoxic effects on neurons. When inflammation associated with degranulation is severe and extends deeply into the muscular layer or around nerves, axonal necrosis may occur, reducing neural conduction efficiency and impairing neurotransmitter transmission, which may lead to ineffective peristalsis or failed propulsion. MBP is toxic to mammalian cells through disruption of the lipid bilayer (58). ECP and EDN possess ribonuclease activity that is toxic to neurons (59). In recent years, eosinophils have also been shown to possess antigen-presenting and immunoregulatory functions, which may contribute directly or indirectly to neuronal injury (60).

Eosinophil-derived MBP can also interfere with neural-to-smooth muscle signaling by antagonizing M2 muscarinic receptors (61). M2 muscarinic receptors are located on parasympathetic nerve terminals within the myenteric plexus and normally function as inhibitory autoreceptors. When activated, they reduce the release of the excitatory neurotransmitter ACh and thereby help maintain the balance between excitation and inhibition. Antagonism of these receptors by MBP enhances parasympathetic excitation and markedly increases ACh release from nerve terminals. The increased ACh then binds to muscarinic receptors on smooth muscle, increasing the likelihood of exaggerated contraction or hyperresponsiveness. Severe disruption of the excitatory–inhibitory balance in the esophageal motor system may therefore produce a variety of manometric abnormalities, including absent contractility, increased distal contractile amplitude, spastic or hypercontractile contractions, and even achalasia-like changes (62).

In addition, Yadavalli et al. (47) found in both patients with EoE and mouse models that eosinophils and mast cells co-accumulate in the esophageal muscularis mucosae. Both cell types are recruited to the vicinity of neural cells within the esophageal wall in response to neuron-derived VIP and jointly contribute to esophageal remodeling and motor dysfunction. *In vitro*, Ogasawara et al. (63) found that specific peptide fragments generated by tryptase-mediated cleavage of MBP and ECP can, in turn, activate mast cells and promote their degranulation, thereby forming a positive-feedback loop that may aggravate impaired esophageal relaxation. These findings suggest that interactions among immune cells may amplify neural injury.

### 5.3 T cells damage inhibitory neurons and impair LES relaxation

In the healthy adult esophagus, T-cell distribution exhibits clear layer specificity. T cells are abundant in the epithelium, vascular papillae, and lamina propria but are less abundant in the muscularis mucosae and external muscle layer. CD4^+^ helper T cells predominate in the mucosa, whereas CD8^+^ cytotoxic T cells are scattered throughout multiple layers (33), where they participate in immune surveillance (64).

Following recognition of antigenic stimuli, these T cells differentiate into distinct subsets according to the local cytokine environment and release corresponding effector cytokines. Regulation of esophageal motility depends on coordination between the central and peripheral nervous systems, and T lymphocytes can modulate this network through several mechanisms, thereby contributing to different patterns of motility dysfunction.

#### 5.3.1 Cooperative infiltration of tissue-resident memory T cells and macrophages affects esophageal motility

Current esophagus-specific evidence suggests that T lymphocytes primarily influence esophageal smooth muscle indirectly by targeting myenteric neurons, a mechanism that has been relatively well documented in AC. Facco et al. (65) found extensive infiltration of CD3^+^CD8^+^ T cells in the LES myenteric plexus of patients with AC, with significantly higher cell numbers than in controls. Liu et al. (66) further examined LES tissue from patients with AC and found marked expansion of ZNF683^+^ CD8^+^ tissue-resident memory T cells and XCL1^+^ CD4^+^ tissue-resident memory T cells (T_RM), which localized around the myenteric plexus and exhibited an inflammatory phenotype. C1QC^+^ macrophages were also expanded and displayed a microglia-like neurodegenerative phenotype. Ligand–receptor interactions were identified between these two cell populations, suggesting that they may amplify local inflammatory responses and form a positive-feedback loop that continuously drives ganglionitis. This process may contribute to the damage or loss of myenteric neurons, resulting in loss of LES innervation, impaired relaxation, and loss of esophageal peristalsis, which clinically manifests as dysphagia and other symptoms of AC.

#### 5.3.2 Helper T-cell subsets influence motility through the release of pro-inflammatory cytokines

Multiple cytokines released by T cells can functionally modulate the enteric nervous system. Furuzawa-Carballeda et al. (67, 68) found significant increases in pro-inflammatory Th22, Th17, Th2, and Th1 subsets in the LES muscular layer of patients with AC, together with an increase in regulatory T cells (Tregs), indicating a T-cell-dominated adaptive immune response. These immune cells localized around the myenteric plexus and released large amounts of pro-inflammatory cytokines, including IL-22, IL-17A, and IFN-γ, which may contribute to myenteric plexus injury. Diseased tissue also exhibited fibrosis and increased MMP-9 expression, suggesting long-standing inflammation accompanied by tissue remodeling and substantial neuronal loss. In areas corresponding to inflammatory infiltration, reductions in VIP-positive inhibitory ganglion cells and ICCs were also observed, indicating impaired inhibitory innervation that may be associated with defective LES relaxation and loss of esophageal body peristalsis.

Ikeda et al. (69) found markedly increased levels of Th17-associated inflammatory cytokines in esophageal tissues from patients with AC, together with reduced phosphorylation of MLC20. These two abnormalities were linked through downregulation of CPI-17, providing a possible explanation for the clinical phenotype of impaired LES relaxation combined with absent esophageal peristalsis.

### 5.4 Macrophage polarization imbalance and C1QC^+^ macrophage complement-mediated pruning synergistically damage inhibitory neurons

Macrophages are essential components of the innate immune system and play critical roles in inflammation and host defense (70). They exhibit considerable plasticity and can differentiate into classically activated macrophages (M1) or alternatively activated macrophages (M2), depending on the local microenvironment (71). In the normal esophagus, macrophages are enriched in the vascular papillae, lamina propria, and muscular layer (33). The M1 phenotype is pro-inflammatory and expresses high levels of cytokines, including IL-1β, IL-6, IL-12, IL-23, and TNF-α. In contrast, the M2 phenotype contributes to the repair and remodeling of damaged tissues and can secrete anti-inflammatory cytokines, such as IL-10 and TGF-β, thereby promoting tissue repair (72). MMφ are located around the myenteric plexus, in close proximity to ICCs and enteric neurons, and are generally considered to exhibit an anti-inflammatory, M2-like phenotype (73, 74). Their survival depends on growth factors secreted by myenteric neurons (75, 76), and they contribute to tissue tolerance that protects myenteric neurons from loss during secondary infection (77). The activation state of MMφ can influence the functions of smooth muscle cells, ICCs, and neurons (3).

Qian et al. (78) found that M1 macrophage levels were significantly increased in the muscular layer of patients with AC, with a higher proportion of M1/MMφ, a lower proportion of M2/MMφ, an increased M1/M2 ratio, and a marked reduction in ICC numbers. Correlation analyses revealed significant associations among macrophage subsets, ICCs, and glial cells. Decreased M2/MMφ levels were accompanied by marked ICC loss and trends toward reductions in S-100β-positive glial cells and nNOS-positive inhibitory neurons. These findings suggest that M1 macrophages may reduce ICC and glial cell populations through their pro-inflammatory functions, including the secretion of IL-6 and TNF-α. In contrast, M2 macrophages may protect the neuronal microenvironment by maintaining glial functions, including heme oxygenase-1 (HO-1)-related pathways, thereby preserving neural conduction and protecting ICCs against oxidative injury. Altered macrophage polarization may therefore change the cellular composition of the esophageal myenteric plexus, resulting in weakened inhibitory input and impaired smooth muscle relaxation.

This process is likely related to the relative proportions of different macrophage polarization states, macrophage-derived cytokines and chemokines, and interactions with other inflammatory cells, including mast cells and eosinophils. By affecting the functions of ICCs and glial cells, these immune components may interfere with neural signal transmission. M1 macrophages may injure nNOS-positive nitrergic neurons, ICCs, and glial cells through inflammatory mechanisms, thereby contributing to impaired LES relaxation. M2 macrophages may exert protective effects, and a reduction in their number may aggravate such injury. Thus, an imbalance in macrophage polarization, particularly an increased M1/M2 ratio, may represent an important step leading to defective LES relaxation and loss of esophageal peristalsis.

Groneberg et al. (79) showed that genetic deletion of NO-sensitive guanylyl cyclase (NO-GC) in the mouse LES completely abolished NO-mediated NANC relaxation, indicating that NO-GC is essential for this pathway. After diffusing into smooth muscle cells, NO activates soluble guanylyl cyclase (sGC), increases intracellular cyclic guanosine monophosphate (cGMP) levels, activates protein kinase G (PKG), reduces intracellular Ca^2+^ levels, and thereby induces muscle relaxation (80).

Liu et al. (66) found that a C1QC^+^ macrophage subpopulation was significantly enriched in esophageal tissue from patients with AC. Further analysis showed that these cells closely surrounded the esophageal myenteric plexus, with some directly infiltrating the plexus. These macrophages highly expressed genes characteristic of central nervous system microglia and exhibited a dysfunctional phenotype associated with neurodegeneration. Pathways related to neurodegenerative diseases, such as Alzheimer’s and Parkinson’s diseases, were enriched, with downregulation of homeostasis-associated genes such as CST3, JUN, and EGR1 and upregulation of disease-associated genes such as APOE, TREM2, and LGALS3. They also highly expressed complement components, including C1QA, C1QB, and C1QC, and showed enrichment of pathways related to synaptic pruning, lysosomal function, and phagocytosis. These changes may interfere with the enteric nervous system, resulting in loss of immune homeostasis and neuronal injury. By disrupting networks involving neurons, glial cells, and ICCs, C1QC^+^ macrophages may ultimately impair the coordination of LES relaxation and esophageal peristalsis.

## 6 Neuropeptides derived from sensory nerve endings

When esophageal nociceptors are activated by acid, capsaicin, intense mechanical stimulation, or inflammatory mediators, sensory nerve endings belonging to extrinsic spinal and vagal afferent pathways are activated (81). On the one hand, these signals are transmitted orthodromically to the central nervous system, where they generate pain perception. On the other hand, SP and calcitonin gene-related peptide (CGRP) can be released locally through axon reflexes involving antidromic or collateral conduction. As key local mediators linking nerves, immune cells, and smooth muscle, these neuropeptides exert bidirectional effects on esophageal motility.

### 6.1 SP enhances cholinergic transmission and directly activates NK2 receptors to promote esophageal contraction

#### 6.1.1 Localization and co-release of SP in the myenteric plexus

SP in the esophagus is predominantly localized to neural structures. Crist et al. (82) identified an excitatory neural pathway in opossum esophageal longitudinal muscle in which ACh and SP coexist. In this pathway, ACh initiates contraction, whereas SP contributes to maintaining the duration of contraction.

Morishita et al. (83) examined the rat esophagus and LES and found that SP-positive neurons and fibers in the myenteric plexus exhibited a decreasing rostrocaudal distribution, suggesting a role in distal esophageal peristaltic propulsion. Most SP-containing neurons were ChAT-positive, whereas only a small proportion were NOS-positive. SP-positive nerve fibers were widely distributed in the muscularis mucosae, LES, and perivascular regions. In contrast, very few SP-positive nerve terminals were detected at the motor endplates of striated muscle, and these terminals frequently coexpressed CGRP. Retrograde tracing revealed numerous SP/CGRP double-positive neurons in the dorsal root and nodose ganglia that projected to the esophagus. In contrast, retrogradely labeled neurons in the dorsal motor nucleus of the vagus and nucleus ambiguus were ChAT-positive but did not express SP, suggesting that SP-containing nerve terminals at motor endplates may originate primarily from extrinsic sensory neurons.

Taken together, intrinsic SP-containing esophageal neurons may function as motor, interneuronal, and vasomotor neurons involved in smooth muscle regulation, local neural transmission, and blood flow control, respectively. The small number of SP-positive terminals at motor endplates may originate from extrinsic sensory neurons and participate in the sensory and motor regulation of striated muscle. Functionally, SP is often co-released with ACh from the same excitatory motor neuron. Through synergistic actions, these transmitters enhance cholinergic transmission and prolong contraction, thereby playing an important role in the regulation of neurogenic esophageal contraction. Collectively, these findings indicate that SP acts as a cotransmitter of excitatory motor neurons and contributes to neurogenic esophageal contraction by enhancing cholinergic transmission and sustaining the duration of contraction.

#### 6.1.2 Direct effects of SP on smooth muscle

SP belongs to the tachykinin family and acts mainly through NK1, NK2, and NK3 receptors (84). It is closely associated with excitatory efferent pathways involved in gastrointestinal motility. Shiina et al. (85) examined the middle thoracic esophagus of rats and found that, after tetrodotoxin (TTX) was used to block voltage-gated sodium channels, SP still induced marked contraction of longitudinal smooth muscle in the esophageal muscularis mucosae. This response was not affected by atropine, indicating that the contractile effect of SP does not depend on neural impulse conduction or cholinergic interneurons but instead results from a direct action on muscle tissue.

The direct contractile effect of SP on esophageal smooth muscle has also been demonstrated in human tissue. Independent of synaptic transmission (86), SP can directly activate NK receptors expressed on esophageal smooth muscle and induce circular muscle contraction, thereby increasing local resistance and intraluminal tension and affecting propulsive efficiency and contractile coordination. Receptor expression in the human LES complex further supports the excitatory role of SP. Zhang et al. (87) examined tachykinin receptor expression in different muscle fiber types of the human LES and found that NK2 was the predominant tachykinin receptor expressed in esophageal sphincter tissue and mediated the major response to tachykinins. Kovac et al. (88) further demonstrated that NK2 receptors are the principal targets mediating tachykinin-induced contraction in human esophageal smooth muscle cells. Functional studies of SP showed that its contractile effects on human esophageal smooth muscle are mediated predominantly through NK2 receptors, with smaller contributions from NK1 and NK3 receptors. Human tissue experiments by Gao et al. (89) further confirmed that SP induces contraction of human esophageal smooth muscle, including both clasp and sling fibers.

### 6.2 CGRP promotes LES hypotonia and facilitates relaxation through three mechanisms while attenuating the propulsive force of esophageal peristalsis

Unlike SP, CGRP does not primarily promote contraction. Instead, it acts at multiple levels to shift the distal esophagus, particularly the LES, toward a state of reduced tone and increased susceptibility to relaxation, while also weakening the propulsive force generated by esophageal body contractions. Its overall effects on esophageal motility may therefore reflect the combined actions of motor neuron neuromodulation, local reflex regulation through sensory nerve endings, and direct effects of circulating or paracrine CGRP on smooth muscle.

Rattan et al. (90) demonstrated an inhibitory effect of CGRP on the LES in opossums. CGRP induced dose-dependent LES relaxation, which was partially antagonized by TTX. Based on these findings, the authors proposed a dual mechanism of CGRP action. At higher concentrations, CGRP may act directly on sphincter smooth muscle, whereas at lower concentrations, it may act on NANC inhibitory neurons in the myenteric plexus and indirectly induce smooth muscle relaxation by promoting the release of inhibitory neurotransmitters. This classic study therefore provided evidence for a neuronally mediated component of CGRP signaling.

The investigators also electrically stimulated the superior laryngeal nerve to evoke swallowing reflexes and stimulated vagal efferent fibers to induce esophageal peristalsis, thereby allowing assessment of the inhibitory effects of CGRP on contraction. CGRP inhibited swallow-induced contractions more strongly than contractions evoked by direct vagal stimulation, suggesting that CGRP may act at both central and peripheral sites. In contrast, Uc et al. (91) found in opossums that CGRP-induced LES relaxation was mediated mainly by a direct action on smooth muscle and did not require activation of intrinsic neurons or the NO pathway. Under *ex vivo* conditions, CGRP directly activated CGRP receptors on LES smooth muscle to induce relaxation, and this response was not affected by TTX or NOS inhibitors. CGRP also directly modulated the off-response of the esophageal body, reducing contraction amplitude and prolonging latency, whereas a CGRP antagonist produced the opposite effect. These findings suggest that endogenous CGRP may be tonically released and participate in the regulation of esophageal smooth muscle tone and the timing of contraction.

Recent studies have provided a new perspective on the role of CGRP in esophageal motor regulation.

Kuramoto et al. (92) found that, in addition to the previously recognized vagal cholinergic motor innervation arising from the compact nucleus ambiguus in the medulla (ChAT/CGRP-positive) and intrinsic NOS-positive innervation from the myenteric plexus, a small proportion of motor endplates on rat esophageal striated muscle fibers also received CGRP-positive sensory nerve terminals. These sensory terminals coexpressed SP and TRPV1, suggesting that some esophageal striated muscle fibers exhibit a triple-innervation arrangement in which the same motor endplate region is influenced by vagal motor fibers, intramural esophageal neurons, and extrinsic SP/CGRP/TRPV1-positive sensory terminals. Retrograde tracing further demonstrated that these CGRP/SP/TRPV1-positive sensory nerve endings originated from peripheral vagal ganglia, including the nodose ganglion (NG), and/or the T10 dorsal root ganglion (T10 DRG). The investigators proposed that TRPV1 channels on sensory endings may detect hydrogen ions derived from acetic acid generated during the breakdown of ACh released from vagal motor terminals, as well as NO released from intrinsic nitrergic neurons. These chemical signals at motor endplates could then be transmitted through sensory nerves to the central nervous system and participate in local reflex regulation of esophageal motor function.

The vagus nerve may also directly innervate esophageal smooth muscle through CGRP-containing fibers. McGovern et al. (93) performed retrograde tracing of vagal motor neurons projecting to the guinea pig esophagus and found that esophagus-projecting vagal motor neurons were located in the rostral compact region of the nucleus ambiguus. Nearly all were cholinergic, and more than 70% also expressed CGRP.

A combined retrograde tracing and immunohistochemical study in rats (94) showed that, following injection of fluorescent tracers into the distal esophagus and cardia, approximately 93.4% of retrogradely labeled neurons in the compact nucleus ambiguus were bilaterally CGRP-immunoreactive, whereas no CGRP-positive labeled neurons were detected in the dorsal motor nucleus of the vagus. These CGRP-positive vagal motor fibers arising from the nucleus ambiguus not only innervate striated muscle in the upper esophagus, as previously recognized, but also project directly to the smooth muscle layer of the distal esophagus and cardia, where they form free nerve endings within the longitudinal and circular muscle layers. Based on these anatomical findings, CGRP released from nucleus ambiguus-derived neurons may act through these free nerve endings on smooth muscle to participate in the regulation of local contraction or relaxation. This suggests that CGRP-positive efferent fibers arising from the nucleus ambiguus may constitute a neural pathway for direct vagal efferent control of smooth muscle in the distal esophagus and cardia.

## 7 Esophageal microbiota regulate esophageal motility and sphincter function through multiple pathways

The esophagus is not a sterile environment and contains distinct microbial community patterns associated with different disease states. Based on composition (95, 96), the esophageal microbiota can be classified into type I and type II communities. Type I microbiota are typically present in the normal esophagus and are composed predominantly of Gram-positive bacteria, with Streptococcus as the major taxon. In contrast, type II microbiota contain a higher proportion of Gram-negative anaerobic or microaerophilic bacteria and are associated with esophageal diseases, including GERD, RE, Barrett’s esophagus, and esophageal adenocarcinoma.

Wong et al. (97) found that specific types of esophageal motility dysfunction in patients with GERD were closely associated with distinct microbial alterations. For example, patients with ineffective esophageal motility and absent contraction reserve showed reduced abundances of Prevotella and Veillonella and enrichment of Fusobacterium nucleatum, suggesting a possible association between changes in the esophageal microbiota and motility disorders. At the mechanistic level, studies have shown that germ-free mice have lower basal vagal activity (98). When these mice are recolonized with a normal microbiota, vagal tone is restored, suggesting that the microbiota may contribute to the maintenance of normal vagal function.

Immune activation represents one of the major mechanisms through which the microbiota may influence esophageal motility. Released inflammatory mediators can directly or indirectly damage neurons innervating esophageal muscles or interfere with smooth muscle contractile proteins, resulting in abnormal contraction or impaired relaxation. Microorganisms colonizing the esophageal mucosal surface may activate local immune responses through their structural components and thereby influence the function and survival of neurons within the esophageal myenteric plexus, ultimately affecting esophageal motility.

Geng et al. (99) found that patients with AC had reduced esophageal microbial diversity and a predominance of Gram-negative bacteria corresponding to a type II microbial community, together with enrichment of lipopolysaccharide (LPS) biosynthesis pathways. At the same time, the number of esophageal myenteric neurons was markedly reduced. These neurons include key inhibitory neurons responsible for smooth muscle relaxation. In subsequent experiments, long-term administration of ampicillin in the drinking water of mice induced esophageal dysbiosis resembling that observed in patients. These mice exhibited a significant reduction in myenteric neuron numbers, increased LES pressure, decreased relaxation responses to inhibitory signals, increased macrophage accumulation in the esophageal lamina propria, activation of the TLR4–MYD88–NF-κB inflammatory pathway, and increased production of the pro-inflammatory cytokine TNF-α.

Mechanistic studies suggested that LPS derived from Gram-negative bacteria is recognized by Toll-like receptor 4 (TLR4) on macrophages, leading to activation of the inflammatory pathway described above and polarization of macrophages toward a pro-inflammatory phenotype. Inflammatory cytokines released by these macrophages, including TNF-α, IL-1β, and IL-6, may cross the mucosal barrier and act on the myenteric plexus, inducing neuronal injury and apoptosis, particularly in inhibitory nitrergic neurons. Loss of inhibitory neuronal function may subsequently impair LES relaxation and reduce the coordination of esophageal peristalsis.

Microbial dysbiosis may also induce excessive expression of inducible nitric oxide synthase (iNOS). Using an opossum model, Fan et al. (100) found that LPS significantly upregulated iNOS protein and mRNA expression in the LES and increased nNOS-dependent NO release. These changes produced a dose-dependent reduction in basal LES tone and markedly enhanced NANC nerve-mediated relaxation in the myenteric plexus. These effects were effectively blocked by a selective iNOS inhibitor. These findings suggest that NANC neurons in the myenteric plexus regulate NO release through nNOS and interact with induced iNOS expression, together contributing to LPS-induced gastrointestinal sphincter dysfunction.

In addition to these neural injury pathways, other studies have identified mechanisms through which the esophageal microbiota may regulate esophageal smooth muscle directly or indirectly. In patients with AC, Th17-associated cytokines, such as IL-17A and IL-22, are markedly upregulated and closely associated with changes in specific microbial taxa, including Actinomyces and Dialister (69). These alterations are accompanied by a significant reduction in MLC20 phosphorylation in esophageal smooth muscle, which may weaken contractile force.

## 8 Clinical implications and therapeutic strategies

The introduction of high-resolution esophageal manometry has provided a more refined approach to the clinical assessment of esophageal motor function. Densely spaced pressure sensors allow detailed characterization of the timing, strength, and propagation patterns of contractions in the esophageal body and sphincters. According to the Chicago Classification (101), motility disorders can be categorized into distinct entities, including achalasia, distal esophageal spasm, hypercontractile esophagus, ineffective esophageal motility, and related disorders. These classifications are highly valuable for clinical diagnosis but also have clear limitations. Manometry primarily reflects the final mechanical output of muscle contraction and cannot directly determine whether an abnormality originates from neural signaling or from the muscle itself. Consequently, although some patients can be clearly assigned to a manometric subtype, the underlying etiology may remain uncertain. Treatment therefore often remains directed toward the contractile abnormality itself, which may result in only transient efficacy and frequent recurrence, highlighting the need for more precise targeted interventions.

We propose that an important reason for this limitation is that the dominant role of neural innervation in esophageal motility has long been underestimated. Neural control is essential for the regulation of esophageal motor function, and the integrity and coordination of neural networks ensure the orderly propagation of peristaltic waves and the timely opening and closure of the sphincters. Once neural regulation is disrupted, whether through loss of inhibitory or excitatory neurons or through abnormal neural transmission, motor commands can no longer be transmitted appropriately to the musculature, resulting in motility dysfunction. At the same time, mucosal sensing is also indispensable. Following mucosal injury, signals must be transmitted through neural pathways to the musculature before corresponding motor disturbances can be generated.

With increasing understanding of the neural mechanisms governing esophageal motility, therapeutic strategies have begun to expand. Traditional treatments mainly target smooth muscle itself, including smooth muscle relaxants such as calcium channel blockers and nitrates, or mechanically relieve outflow obstruction through procedures such as peroral endoscopic myotomy (POEM). More recently, neuromodulatory approaches have attracted increasing attention. Techniques such as transcutaneous electrical stimulation (102) and electroacupuncture (103) may modulate vagal tone and help restore more physiological patterns of esophageal motility, with the potential to improve therapeutic efficacy while reducing adverse effects and recurrence.

In addition, a recent animal study (104) found that viral infection caused increased LES pressure and motor dysfunction, accompanied by a marked reduction in the number of myenteric plexus neurons. Mechanistic analyses showed that the expression of proteins associated with cell survival and functional maintenance, including Transgelin and HSPB1, was significantly reduced after infection, whereas ferroptosis-related pathways were activated. This was characterized by upregulation of the ferroptosis-promoting factors COX-2 and ACSL4 and downregulation of the key anti-ferroptotic molecules GPX4 and FTH1. Administration of an exogenous hydrogen sulfide donor attenuated these abnormalities by inhibiting ferroptosis. These findings suggest that ferroptosis-mediated neural injury may represent another potential mechanism underlying esophageal motility disorders and may provide new molecular targets for neuromodulatory intervention.

Regarding neuroimmune networks, multiple studies in recent years have demonstrated that natural products can act on neuroimmune nodes associated with esophageal motility. Berberine is one of the more extensively studied monomeric components derived from natural products. In terms of immune regulation, berberine combined with evodiamine can act synergistically in the esophageal tissue of rats with GERD and, through TAS2R38/TRPV1-mediated MAPK/NF-κB signaling, inhibit the phosphorylation of p65, JNK, and ERK, regulate macrophage polarization, and alleviate local esophageal inflammation (105). In terms of motility, experiments using isolated LES muscle strips suggest that berberine can induce a relaxant effect through potassium channel activation (106). The dual potential of berberine for immune regulation and myogenic relaxation plays an important role in the traditional Chinese medicine formula Zuojin Pill. However, these two lines of evidence were derived from different experimental systems, and studies directly linking immunoregulatory effects with esophageal motility endpoints are still lacking. Compound formulas, such as Xuanfu Daizhe Decoction, exhibit both local esophageal immunoregulatory effects and improvement in esophageal motility. In a rat model of RE (107), Xuanfu Daizhe Decoction was found to reduce TREM-1 and CD68 expression in esophageal tissue and promote macrophage polarization toward the M2 phenotype by inhibiting STAT1/TREM-1 signaling, thereby alleviating esophageal inflammatory responses. Regarding motility regulation, observations in patients with GERD (108) showed that esophageal motility-related parameters improved after treatment with modified Xuanfu Daizhe Decoction, LES- and UES-related parameters increased, the success rate of wet swallows increased, and reflux duration was shortened. Experimental and clinical evidence has thus separately demonstrated immune regulation and motility improvement for the same formula, providing a basis for further investigation of the relationship between the immune microenvironment and esophageal neuromuscular function. Although studies directly linking immunoregulatory effects with esophageal motility endpoints remain lacking, the above findings suggest that natural products may indirectly influence the esophageal motility regulatory network by acting on upstream nodes of neuroimmune pathways. This possibility warrants further validation in future studies of esophageal motility disorders and also provides new potential therapeutic targets.

## 9 Conclusion

The development and progression of esophageal motility disorders result from disruption of the complex coordination among mucosal sensing, neural innervation, and muscular contraction. Propulsive movement through the esophagus from the proximal to the distal segment depends on precise signal transmission among the central nervous system, peripheral nerves, intramural esophageal neural networks, and esophageal smooth muscle, while also being modulated by feedback from mucosal sensory receptors. The generation and transmission of neural commands, the ability of the musculature to respond to these commands, and mucosal sensing and responses to stimulation together form an integrated network that regulates esophageal motility. Dysfunction at any level of this network may disrupt this balance and contribute to disease.

Local neural control of the esophagus, including the regulatory mechanisms of local reflex arcs and the specific roles of the intramural nervous system in maintaining esophageal function, has received relatively limited attention, and both experimental and clinically oriented mechanistic studies remain scarce. Moreover, the sequence of activation, temporal relationships, and interactions between local regulatory pathways and long reflex pathways mediated through the central nervous system under physiological and pathological conditions remain poorly defined. Consequently, the overall mechanisms of neural regulation of esophageal function are still incompletely understood, and many critical questions require further investigation.

It should be emphasized that a substantial proportion of the evidence discussed in this review regarding neural regulation of the esophagus is derived from animal models, including opossums, cats, mice, guinea pigs, and pigs. These species differ from humans in esophageal neuroanatomy, receptor subtype distribution, and neurotransmitter regulation. Caution is therefore required when extrapolating these mechanisms to human esophageal neuromuscular physiology, particularly when extending these findings to human achalasia or GERD. Although we have incorporated evidence from human esophageal tissues and clinical specimens wherever possible, some mechanistic links remain based predominantly on animal experiments because of limitations in the available literature. The correspondence between animal findings and human physiology therefore requires further clarification.

Similarly, current human evidence regarding the relationship between the esophageal microbiota and motility disorders is largely based on cross-sectional observations. Whether microbial dysbiosis is a cause of esophageal motility dysfunction or a consequence of altered esophageal physiology remains unresolved. Further studies based on human esophageal tissues and *in vivo* human-relevant models are therefore required to determine the actual contribution of these mechanisms in humans.

In summary, this review delineates the close interactions among the mucosa, nervous system, and musculature in the regulation of esophageal motility. These findings provide new insights into the neural mechanisms underlying esophageal motility disorders and support further exploration of therapeutic strategies targeting neural regulation.
